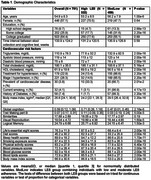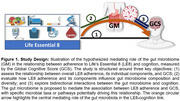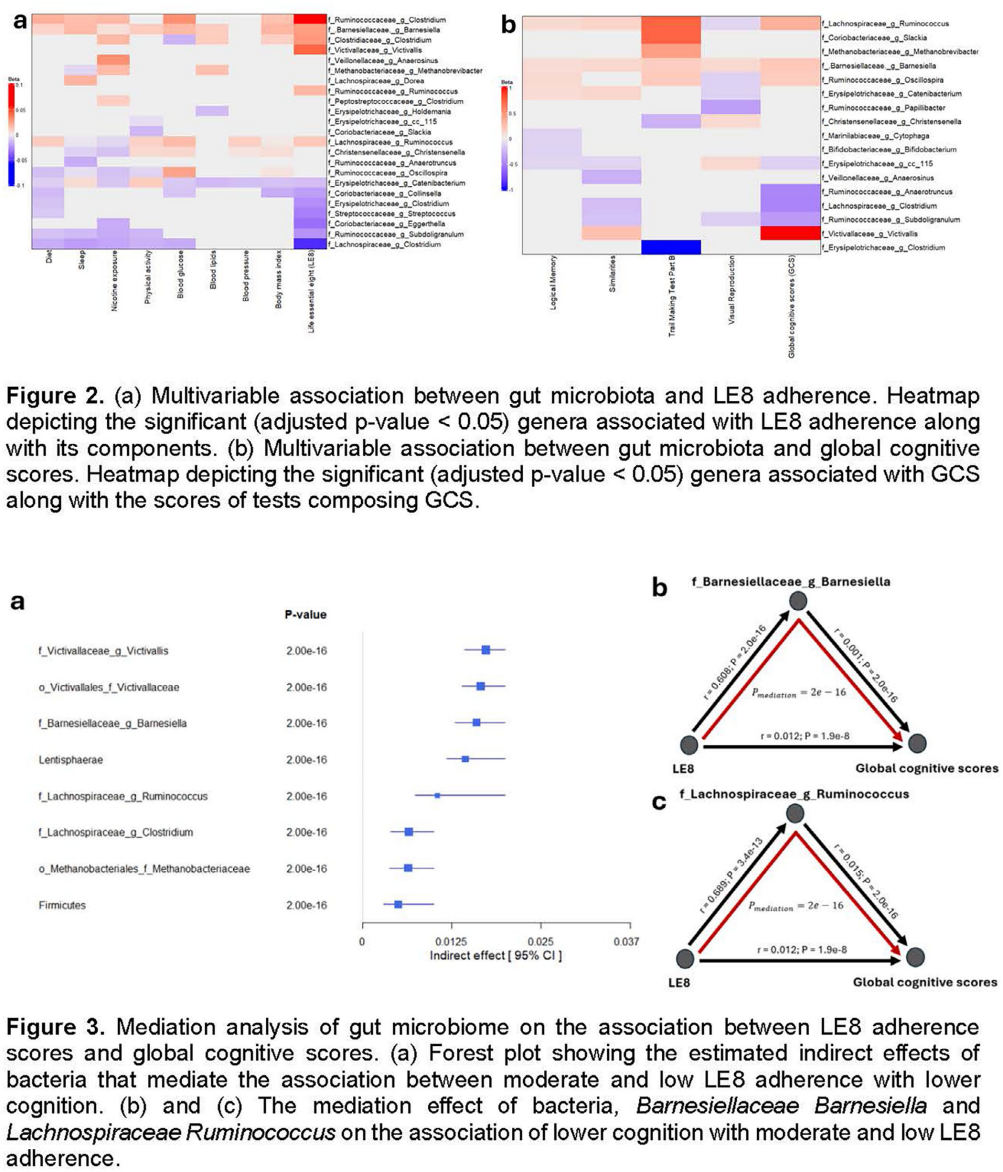# Gut Microbiome Diversity as a Mediator Between Life's Essential 8 Adherence and Cognitive Function

**DOI:** 10.1002/alz70860_103169

**Published:** 2025-12-23

**Authors:** Yannick Joel Wadop Ngouongo, Jazmyn A Muhammad, Rebecca Bernal, Claudia L. Satizabal, Alexa S Beiser, Vasan S. Ramachandran, Tiffany F. Kautz, Sudha Seshadri, Jayandra Jung Himali, Bernard Fongang

**Affiliations:** ^1^ Glenn Biggs Institute for Neurodegenerative Diseases, University of Texas Health Science Center at San Antonio, San Antonio, TX, USA; ^2^ Glenn Biggs Institute for Alzheimer's & Neurodegenerative Diseases, The University of Texas Health Science Center at San Antonio, San Antonio, TX, USA; ^3^ Glenn Biggs Institute for Alzheimer's & Neurodegenerative Diseases, University of Texas Health San Antonio, San Antonio, TX, USA; ^4^ Framingham Heart Study, Framingham, Boston, MA, USA; ^5^ Department of Population Health Sciences, University of Texas Health Science Center at San Antonio, San Antonio, TX, USA; ^6^ Department of Neurology, Boston University School of Medicine, Boston, MA, USA; ^7^ Boston University School of Public Health, Boston, MA, USA; ^8^ Boston University School of Medicine, Boston, MA, USA; ^9^ The Framingham Heart Study, Framingham, MA, USA; ^10^ Boston University Chobanian & Avedisian School of Medicine, Boston, MA, USA; ^11^ Department of Medicine, Section of Cardiovascular Medicine, Boston Medical Center, Boston University School of Medicine, Boston, TX, USA; ^12^ Department of Medicine, Section of Preventive Medicine and Epidemiology, Boston University School of Medicine, Boston, MA, USA; ^13^ Department of Epidemiology, Boston University School of Public Health, Boston, MA, USA; ^14^ Boston University's Center for Computing and Data Sciences, Boston, MA, USA; ^15^ The University of Texas School of Public Health in San Antonio, San Antonio, TX, USA; ^16^ The Long School of Medicine, University of Texas Health Science Center, San Antonio, TX, USA; ^17^ Glenn Biggs Institute for Alzheimer's and Neurodegenerative Diseases, University of Texas Health Science Center, San Antonio, TX, USA; ^18^ Department of Medicine, University of Texas Health Science Center at San Antonio, San Antonio, TX, USA; ^19^ Glenn Biggs Institute for Alzheimer's & Neurodegenerative Diseases, University of Texas Health Science Center, San Antonio, TX, USA; ^20^ Department of Neurology, University of Texas Health Sciences Center, San Antonio, TX, USA, San Antonio, TX, USA; ^21^ Department of Population Health Sciences, University of Texas Health Sciences Center, San Antonio, TX, USA; ^22^ Department of Population Health Sciences, UT Health San Antonio, San Antonio, TX, USA; ^23^ Department of Biostatistics, Boston University School of Public Health, Boston, MA, USA; ^24^ Department of Population Health Sciences, The University of Texas Health Science Center at San Antonio, San Antonio, TX, USA; ^25^ Department of Biochemistry and Structural Biology, The University of Texas Health Science Center at San Antonio, San Antonio, TX, USA

## Abstract

**Background:**

Emerging evidence indicates a complex interplay between cardiovascular health, gut microbiome composition, and cognitive function. Life's Essential 8 (LE8), created by the American Heart Association, encompasses crucial cardiovascular health metrics. This study aimed to explore the relationship between LE8 adherence, gut microbiota, and cognition.

**Method:**

We used stool samples, LE8 metrics, and cognitive assessment measures from a sample of 781 participants (mean age 54.9 years, 57.1% Female) from the Framingham Heart Study (generation3, New Offspring Spouses, and the Omni2 cohorts) at the 3^rd^ examination (2016‐2019). Associations between LE8 adherence, gut microbiome diversity, and cognitive performance were evaluated using multivariable linear regression models, adjusting for potential confounders. Mediation analysis was conducted to explore whether specific bacterial taxa mediated the relationship between LE8 adherence and cognitive performance.

**Result:**

Participants with greater adherence to LE8 demonstrated significantly increased gut microbial diversity (α‐diversity: Chao1, *p* = 0.0014; Shannon, *p* = 0.0071) and distinct microbial compositions (β‐diversity: PERMANOVA *p* = 1e‐4). Higher adherence to LE8 was related to an increased abundance of genera *Barnesiella* and *Ruminococcus*, while reduced abundance of *Clostridium* was associated with higher LE8 adherence. Greater gut microbial diversity (α‐diversity: Chao1, *p* = 0.0012; Shannon, *p* = 0.0066), and beneficial genera like *Oscillospira* correlated with better global cognitive scores (GCS). Taxonomic overlap analyses revealed microbial taxa that simultaneously influence LE8 adherence and cognitive outcomes. Mediation analyses indicated that specific taxa, including *Barnesiella* and *Lentisphaerae*, mediated the link between higher LE8 adherence and better cognitive performance. These taxa could be key modulators in the gut‐brain axis, connecting cardiovascular and brain health. Conversely, higher Clostridium abundance was associated with poorer cognitive performance.

**Conclusion:**

This study highlights the interconnected relationship between cardiovascular health, gut microbiome diversity, and cognitive function. Higher adherence to LE8 was associated with favorable microbial profiles and better cognitive performance, with the gut microbiome serving as a critical mediator. These findings emphasize the importance of integrated lifestyle interventions that address cardiovascular and cognitive health simultaneously. To validate these results and refine therapeutic strategies, future research should prioritize longitudinal studies and randomized controlled trials that explore the causal pathways and clinical applications of these findings.